# Safety and possible effects of low-intensity resistance training associated with partial blood flow restriction in polymyositis and dermatomyositis

**DOI:** 10.1186/s13075-014-0473-5

**Published:** 2014-10-25

**Authors:** Melina Andrade Mattar, Bruno Gualano, Luiz Augusto Perandini, Samuel Katsuyuki Shinjo, Fernanda Rodrigues Lima, Ana Lúcia Sá-Pinto, Hamilton Roschel

**Affiliations:** Division of Rheumatology, School of Medicine, University of São Paulo, São Paulo, Brazil; School of Physical Education and Sports, University of São Paulo, São Paulo, Brazil; Faculdade de Medicina Universidade de São Paulo - Divisão de Reumatologia, Av Dr Arnaldo, 455 - 3° andar - sala 3131 - Cerqueira César, São Paulo, Brazil

## Abstract

**Introduction:**

Our aim was to evaluate the safety and efficacy of a low-intensity resistance training program combined with partial blow flow restriction (BFR training) in a cohort of patients with polymyositis (PM) and dermatomyositis (DM).

**Methods:**

In total, 13 patients with PM and DM completed a 12-week twice a week low-intensity (that is, 30% one-repetition-maximum (1RM)) resistance exercise training program combined with partial blood flow restriction (BFR). Assessments of muscle strength, physical function, quadriceps cross sectional (CSA) area, health-related quality of life, and clinical and laboratory parameters were assessed at baseline and after the intervention.

**Results:**

The BFR training program was effective in increasing the maximal dynamic strength in both the leg-press (19.6%, *P* <0.001) and knee-extension exercises (25.2% *P* <0.001), as well as in the timed-stands (15.1%, *P* <0.001) and timed-up-and-go test (−4.5%, *P* =0.002). Quadriceps CSA was also significantly increased after the intervention (4.57%, *P* =0.01). Similarly, all of the components of the Short Form-36 Health Survey, the Health Assessment Questionnaire scores, and the patient- and physician reported Visual Analogue Scale were significantly improved after training (*P* <0.05). Importantly, no clinical evidence or any other self-reported adverse event were found. Laboratory parameters (creatine kinase and aldolase) were also unchanged (*P* >0.05) after the intervention.

**Conclusions:**

We demonstrated that a 12-week supervised low-intensity resistance training program associated with partial blood flow restriction may be safe and effective in improving muscle strength and function as well as muscle mass and health-related quality of life in patients with PM and DM.

**Trial registration:**

Clinicaltrials.gov NCT01501019. Registered November 29, 2011.

## Introduction

Polymyositis (PM) and dermatomyositis (DM) are part of a group of rare conditions named idiopathic inflammatory myositis (IIM) that are characterized by proximal muscle weakness [1] as well as muscle atrophy, fatigue, myalgia, and impairment in distal lower-limb muscle function, [2,3]. Collectively, these features lead to limitations in activities of daily living and poor quality of life [4,5].

In this regard, physical exercise, specifically resistance exercise training, has been proposed as a safe and effective therapeutic tool to counteract several of the symptoms related to muscle dysfunction in patients with PM and DM [6-8]. In fact, resistance training has been shown to improve isometric peak torque, muscle strength, muscle endurance, functional capacity, and quality of life in PM and DM patients [6,8-12]; however, only one study has investigated the benefits of this exercise modality on increase in muscle mass in PM/DM patients, with the results showing significant improvements in type-II fiber cross-sectional area (CSA) [13].

Importantly, exercise intensities within the 70% to 85% of one-repetition-maximum (1RM) range have been recommended for increases in muscle mass and strength [14]. Alternatively, low-intensity resistance training (for example, 20% to 30% 1RM) combined with partial blood flow restriction (BFR) induces similar gains in muscle mass and strength as compared to conventional high-intensity resistance training in different populations (for example, athletes, healthy young adults, and the elderly) [15-20]. The clinical application of this intervention has the potential to represent an interesting approach to resistance exercise in IIM, as patients are often unable to exercise at high intensities. In fact, BFR training has been previously shown to increase muscle strength, balance, quality of life, and thigh CSA in one patient with inclusion body myositis [21]. However, there are no studies applying BRF training in a cohort of patients with PM/DM patients. Therefore, the aim of the present study was to evaluate the safety and efficacy of a BFR training program in a cohort of patients with PM and DM.

## Methods

### Study design and patients

This prospective, longitudinal, quasi-experimental study was conducted between 2011 and 2013 in São Paulo, Brazil (Clinical Hospital, School of Medicine, University of São Paulo). This study was registered at *clinicaltrials.gov* as NCT01501019.

**Figure 1:**
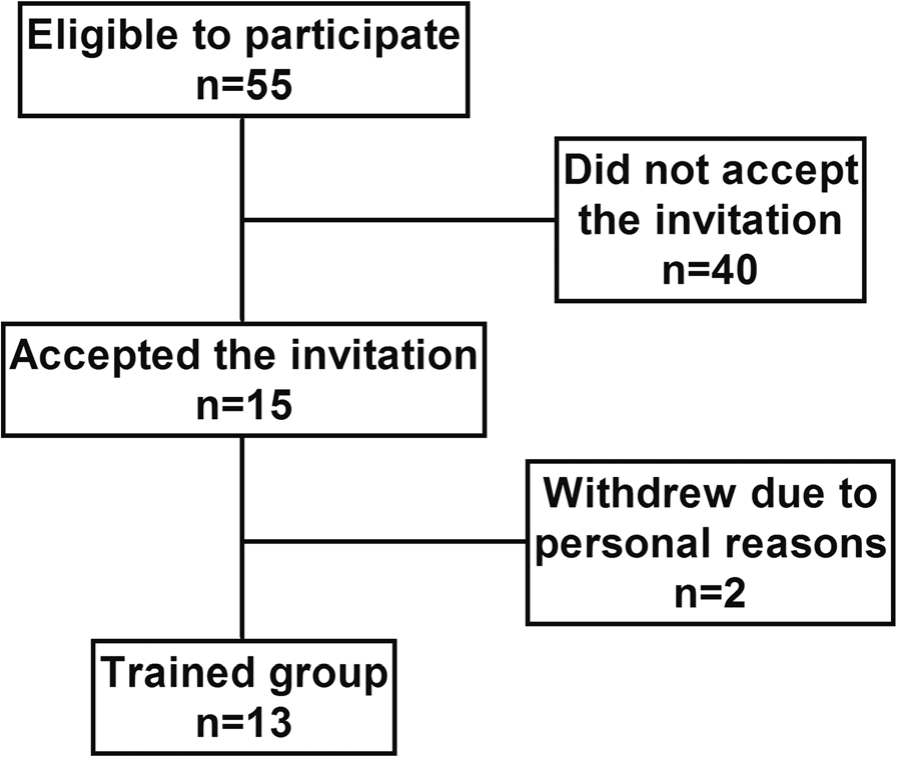
**Flowchart of the protocol for the patients with polymyositis and dermatomyositis.**

Thirty-seven patients with non-active PM and DM from our outpatient clinic were eligible to participate in this trial and only 15 accepted the invitation to take part in the study. Two patients withdrew before the commencement of the study due to personal reasons. Therefore, 13 patients (9 women and 4 men) completed the protocol and were evaluated (age 45.6 ± 8.8 years; BMI 31.0 ± 6.6 kg/m^2^; peak oxygen consumption (VO_2peak_) 23.3 ± 6.9 mL/kg/min). Figure 1 shows the flow chart for the protocol. All of the patients were diagnosed with definite PM/DM according to the Bohan and Peter criteria [22]. Exclusion criteria included: acute or chronic infection; other untreated chronic disease (for example, hepatic, kidney, endocrine and/or cardiac involvement); physical limitations that could preclude the participation in the physical tests and training; and active disease. The patients were not engaged in any form of exercise for at least six months prior to the study, and 12 out of the 13 patients had never had any previous experience with regular exercise. The only exception is one patient who had been enrolled in a resistance exercise routine (two times a week for 3 months) 2.5 years prior to the commencement of the present study. The study was approved by the Local Ethical Committee (School of Medicine, University of São Paulo) and all of the patients signed the written informed consent form. Table 1 shows the patients’ main features and the drug regime.

**Table 1:** **Patients’ characteristics and drug regimen**

**Patients (disease)**	**BMI, kg/m** ^**2**^	**Disease duration, months**	**Current use of immunosuppressant drugs**
I (DM)	30.5	61	AZA
II (DM)	38.6	60	CQ; MTX; CP
III (DM)	38.2	151	None
IV (PM)	27.9	23	AZA
V (DM)	21.4	75	CQ; CP
VI (DM)	26.5	52	AZA; MTX; Prednisone
VII (PM)	31.4	99	MTX 15
VIII (PM)	32.4	26	AZA; MTX
IX (DM)	23.5	82	MTX
X (DM)	27.4	37	HCQ; AZA
XI (PM)	25.2	38	MMF
XII (DM)	43.0	93	AZA
XIII (DM)	37.7	48	AZA
All patients, mean (SD)	31 (6.6)	65 (35)	Not applicable

DM, dermatomyositis; PM, polymyositis; BMI, body mass index; AZA, azathioprine; CQ, chloroquine diphosphate; MTX, methotrexate; CP, cyclosporine; HCQ, hydroxychloroquine sulphate; MMF, mycophenolate mofetil.

At baseline (PRE) and after the intervention (POST), independent assessors evaluated health-related quality of life, muscle strength and function, quadriceps CSA, and laboratory and clinical parameters.

### Exercise training program

The exercise program consisted of a twice-weekly 12-week supervised BFR training program (that is, low-intensity resistance exercises combined with partial blood flow restriction). Exercise sessions were monitored by at least two fitness professionals and were performed in an intrahospital gymnasium (Laboratory of Assessment and Conditioning in Rheumatology, Clinical Hospital, School of Medicine, University of São Paulo, Brazil). The BFR training program was comprised of conventional two-legged leg-press (Nakagym®, São Paulo, Brazil) and knee extension (Nakagym®) exercises. The restriction in blood flow was obtained by placing an air cuff (width 175 mm × length 920 mm) at the inguinal fold of both thighs, as previously described [23]. A partial blood-flow restriction was sustained throughout the entire training sessions (25 to 30 minutes), including the rest intervals and it was released immediately after the end of the training sessions. During the first 4 weeks of training, patients were required to perform four sets of 15 repetitions with 30% 1RM. During the first week, a reduced intensity of 20% 1RM for each exercise was performed. From the fifth week on, exercise volume was increased to five sets of 15 repetitions with 30% 1RM. Exercise load was adjusted every 4 weeks by reassessment of the patients’ 1RM and a 1-minute rest period was allowed between sets throughout the entire training protocol. A member of the research staff monitored the adherence to the exercise program on a session basis. The patients completed an average of 22.6 ± 3 out of the 24 exercise sessions originally planned (94 ± 9% adherence rate).

### Determination of the blood flow restriction pressure

Before the commencement of the training protocol, blood flow restriction pressure was determined. Subjects were asked to comfortably lie supine while a vascular Doppler probe (DV-600, Marted, Ribeirão Preto, SP, Brazil) was placed over the tibial artery to capture its auscultatory pulse. The blood pressure (mmHg) necessary for the complete blood flow restriction (pulse elimination pressure) was determined by attaching a standard blood pressure cuff to the participant’s thigh (inguinal fold region) and then inflating it up to the point in which the auscultatory pulse was interrupted [23]. The cuff pressure utilized during the training protocol was determined as 70% of the predetermined pulse elimination pressure. A mean of 94 ± 13 mmHg of cuff pressure was utilized during the exercise sessions. For visual details on the procedures for the determination of blood flow restriction pressure and administration of the low-resistance training combined with partial blood flow restriction, please refer to a previous description of the methods [23].

### Muscle strength and physical function assessments

The patients underwent two familiarization sessions separated by at least 72 h for their assessment of 1RM. The 1RM tests were performed for the leg-press and knee extension exercises according to previously described criteria [24]. In brief, prior to the 1RM test, two light warm-up sets were performed with 2 minutes interspersed. Then, the patients had up to five attempts to achieve the 1RM load, with a 3-minute interval between attempts. Verbal encouragement was provided and testing sessions were conducted by two experienced researchers.

Physical function was assessed by the timed-stands test (TST) and the timed up-and-go test (TUG). The TST evaluates the maximum number of stand-ups that a subject can perform from a standard-height (that is, 45 cm) armless chair within 30 seconds, whereas TUG evaluates the time that is required for the subject to rise from a standard arm chair, walk towards a line drawn on the floor three meters away, turn, return, and sit back down again. The coefficients of variation (CV) for the muscle function tests were 3.0%, 4.1%, 2.6%, and 1.7% for the 1RM leg press, 1RM knee extension, TST, and TUG, respectively.

### Quadriceps CSA

Magnetic resonance imaging (MRI) was used to obtain quadriceps CSA (Signa LX 9.1, GE Healthcare, Milwaukee, WI, USA). Patients lay in a supine position with their knees extended and legs straight. A bandage was used to restrain leg movements during the test. An initial reference image was obtained to determine the perpendicular distance from the greater trochanter of the femur to the inferior border of the lateral epicondyle of the femur, which was defined as the segment length. Quadriceps CSA was measured at 50% of the segment length with 0.8 cm slices for 3 seconds. The pulse sequence was performed with a view field between 400 and 420 mm, repetition time of 350 milliseconds, echo time from 9 to 11 milliseconds, two signal acquisitions, and matrix of reconstruction of 256 × 256. The quadriceps images were traced in triplicates by a specialized researcher, and their mean values were used for further analysis. The between-measurements CV was 0.6%.

### Quality of life and limitation of daily activities assessments

The short form-36 health survey (SF-36) was used to assess health-related quality of life before and after the BRF training program. A maximal score of 100 indicates the best health condition. The health assessment questionnaire (HAQ) assessed limitation of daily activities with scores ranging from 0 to 3, where the minimal score indicates the best health condition.

### Clinical and laboratory assessments

Clinical and laboratory assessments were performed at baseline and after the exercise training program. Safety analysis included the assessment of laboratory parameters of inflammation (creatine kinase (CK) and aldolase) before the commencement of the training protocol and 48 h after the last training session. Additionally, patients’ and physicians’ global assessment subscales from the visual analog scale (VAS) were also assessed. The patients were advised to report any sort of adverse events and potential disease-flare episodes were monitored throughout the study. Finally, all of the patients were examined by a rheumatologist on a weekly basis throughout the intervention in order to evaluate any possible symptoms of excessive exhaustion, pain, osteoarticular injury, muscle soreness, or any other adverse event.

### Maximal-graded cardiorespiratory test

Before the trial, all of the subjects underwent a maximal graded cardiorespiratory test on a treadmill (Centurion 200, Micromed, Brasilia, Brazil) for VO_2peak_ determination at baseline. VO_2_ and carbon dioxide output were obtained through breath-by-breath sampling and expressed as a 30-second average using an indirect calorimetry system (Cortex - model Metalyzer III B, Leipzig, Germany). Heart rate was continuously recorded at rest, during exercise and at recovery, using a 12-lead electrocardiogram (Ergo PC Elite, InC. Micromed). VO_2peak_ was considered as the average of the final 30 seconds of the test. Attainment of VO_2peak_ was considered to be when two of three criteria were met at the volitional exhaustion: VO_2_ plateau (that is, <150 mL/minute increase between two consecutive stages), heart rate (HR) no less than 10 beats below age-predicted maximal HR, respiratory exchange ratio value above 1.10, and rating of perceived exertion ≥17 of the 15-point Borg scale [25].

### Statistical analysis

Data are expressed as mean ± SD. The paired *t*-test was used to assess possible differences between pre- and post-intervention periods. Effect sizes (ES) for all of the dependent variables were calculated according to a previous description [26]. The Wilcoxon test was applied to non-parametric data (that is, SF-36, HAQ and VAS data). The significance level was previously set at *P* ≤0.05.

## Results

### Muscle strength and physical function

**Figure 2:**
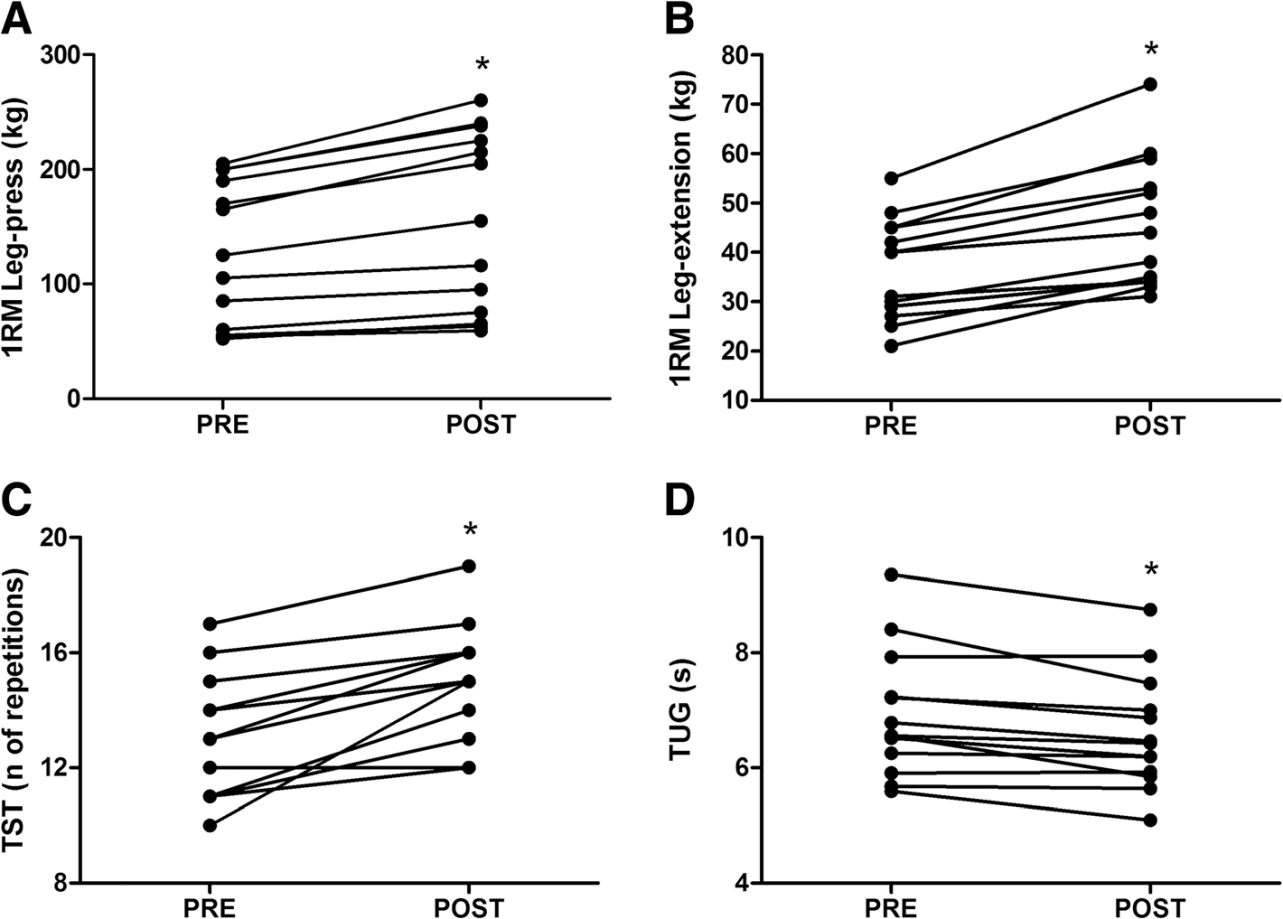
**Muscle strength and physical function data at baseline (PRE) and after 12 weeks of intervention (POST). (A)** Leg-press exercise one-repetition-maximum (1RM) data; **(B)** knee-extension exercise 1RM data; **(C)** timed-stands test (TST) data; **(D)**: timed up-and-go test (TUG) data. *P <0.05 when compared with baseline assessments.

The BFR training program was effective in increasing the 1RM in both the leg-press and the knee-extension exercises as observed by improvements of 19.6% (ES 0.42, *P* <0.001) and 25.2% (ES 0.87, *P* <0.001), respectively. We also observed significant improvements in the TST scores (15.1%; ES 0.87; *P* <0.001) and TUG scores (−4.5%; ES: −0.29; *P* =0.002) after the intervention (Figure 2).

### Quadriceps CSA

**Figure 3:**
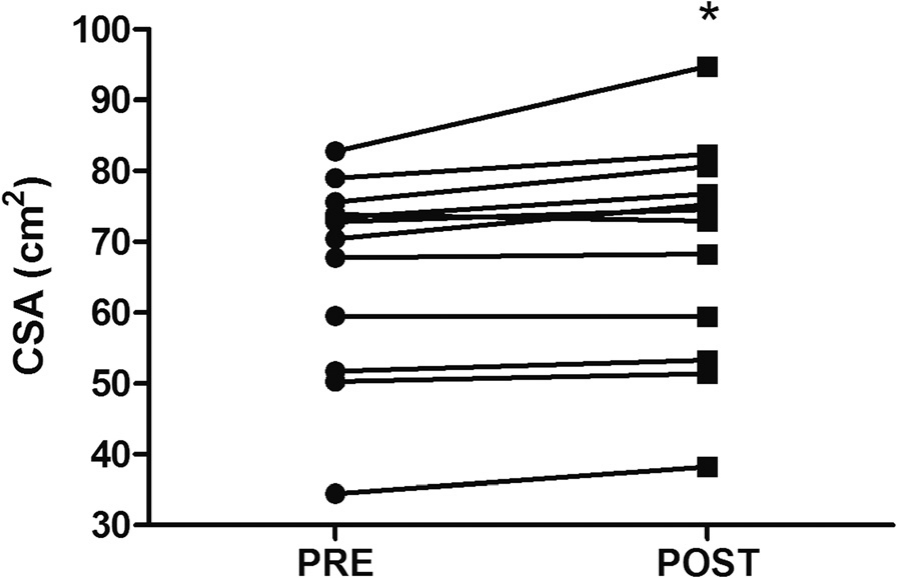
**Quadriceps cross-sectional area (CSA) at baseline (PRE) and after 12 weeks of intervention (POST).** *P <0.05 when compared with baseline assessments.

One patient missed the follow-up analysis for quadriceps CSA, therefore, our data shows results from 12 patients. The quadriceps CSA was significantly increased after the BFR training program (4.57%; ES 0.21; *P* =0.01). Importantly, 10 out of the 12 patients analyzed showed improvements in CSA above that of the CV of the measurement after the intervention. Figure 3 shows the quadriceps CSA data.

### Quality of life and limitation of daily activities assessments

Health-related quality of life measured by the SF-36 was significantly improved in all of its components after the intervention. Similarly, the HAQ scores and both the patient- and physician-reported VAS were also significantly improved after the BFR training program (*P* <0.05 for all of the variables). Table 2 shows the quality of life and limitation of daily activities data. Importantly, no adverse effects from the blood-flow restriction protocol (for example, excessive fatigue or pain) were reported by any of the subjects.

**Table 2:** **Effects of a 12-week supervised BFR training program on health-related quality of life, limitation of daily activities and laboratory parameters in patients with polymyositis and dermatomyositis**

**Variable**	**PRE**	**POST**	**ES**	**%**	***P*** **-value (pre- to post-test)**
SF-36 physical function	57.5 ± 23.2	80.4 ± 11.6	1.03	39.82	0.003*
SF-36 role physical	31.3 ± 42.8	62.5 ± 39.2	0.76	99.68	0.041*
SF-36 bodily pain	59.4 ± 11.4	86.9 ± 14.8	2.52	46.29	0.002*
SF-36 general health	71.4 ± 20.1	83.8 1 ± 5.6	0.65	17.36	0.003*
SF-36 vitality	54.6 ± 20.4	84.2 ± 9.3	1.52	54.21	0.003*
SF-36 social function	65.6 ± 33.7	89.6 ± 17.5	0.74	36.58	0.017*
SF-36 role emotional	27.8 ± 44.6	77.8 ± 38.5	1.17	179.85	0.014*
SF-36 mental health	62.3 ± 20.8	79.0 ± 16.9	0.83	26.80	0.007*
HAQ	1.5 ± 1.9	0.6 ± 0.6	−0.49	−60.00	0.004*
VAS patient	3.4 ± 1.4	1.6 ± 1.2	−1.51	−52.94	0.008*
VAS physician	2.6 ± 1.2	1.2 ± 0.6	−1.21	−53.84	0.004*

PRE (baseline) and POST (after the intervention) values are presented as mean ± SD. BFR, low-intensity resistance training combined with partial blood flow restriction; PM, polymyositis; DM, dermatomyositis; HAQ, health assessment questionnaire; VAS patient, patient-reported visual analog scale; VAS physician, physician-reported visual analog scale. *P <0.05 when compared with baseline assessments.

### Safety analysis and clinical and laboratory assessments

No clinical evidence of excessive fatigue, pain, osteoarticular injury, muscle soreness, or any other self-reported adverse event was found. Laboratory parameters were unchanged after the intervention (CK PRE versus POST, 195 ± 76 versus 224 ± 171 IU/L, respectively; *P* =0.405; aldolase PRE versus POST, 3.9 ± 1.5 versus 4.1 ± 1.6 IU/L; *P* =0.592). The drug regimen (that is, number, type and dose of medications) remained unchanged throughout the study.

## Discussion

This was the first study to investigate the effects of low-intensity resistance training combined with partial BFR in a cohort of PM and DM patients. This exercise mode was shown to be effective in counteracting important symptoms in PM/DM, including muscle weakness, muscle dysfunction, muscle atrophy, and poor health-related quality of life. Of note, no exacerbation of the disease was observed. Collectively, these data provide evidence suggesting that BFR training may be both safe and effective for non-active patients with PM and DM.

The beneficial effects of conventional resistance training in patients with PM and DM have been previously demonstrated [10,11]. Alexanderson *et al*. [10] reported in a pilot study that 12 weeks of home-based resistance training was effective in increasing muscle endurance and quality of life in PM and DM patients, while no adverse effects were observed. In accordance, the same research group [11] demonstrated that a similar training protocol was safe and effective in improving function and quality of life in PM and DM patients with active disease. This findings have been corroborated by others [6,12,27], however, to our knowledge, only one study has evaluated the effects of resistance training on muscle mass in patients with PM and DM [13]. Following a 12-week home-based physical training program, significant increases (25%) in type-II, but not in type-I fiber CSA were reported in these patients.

Importantly, increases in muscle mass and strength usually require high-intensity resistance training, with loads varying between 70% and 85% 1RM [14]. As IIM are characterized by remarkable impairments in muscle strength and function, these patients are often unable to exercise at higher intensities, warranting the need for alternative exercise strategies aiming to increase muscle strength and mass while minimizing exercise intensity. In this context, low-intensity strength training associated with BFR has emerged as an interesting therapeutic tool to these patients, as it has been shown to induce similar gains in muscle strength and mass to those observed after conventional high-intensity resistance training [15-20]. The putative mechanisms for the positive effects of BFR training have been attributed to the augmented metabolic stress induced by the diminished blood flow, which may potentially lead to: i) increased muscle fiber recruitment during exercise [28,29]; ii) elevated systemic hormonal production [30,31]; iii) enhanced muscle protein synthesis via increased phosphorylation of key proteins involved in muscle trophism signaling [32,33].

To our knowledge, no study except for a case report from our group [21] has evaluated the effects of BFR training in IIM. In this regard, we have previously shown the potential of this exercise modality in improving muscle strength and thigh CSA in one patient with inclusion body myositis. These observations were confirmed and extended to PM and DM in the current study.

The BFR training program was also able to significantly improve lower-limb maximal muscle strength and muscle function. Impairment in activities of daily living due to proximal and distal muscle weakness is a prominent feature in PM and DM, warranting great clinical relevance to these findings. Interestingly, these improvements were paralleled by a reduction in the limitations of daily activities (that is, improvement in HAQ scores), and improvements in quality of life as observed by improved scores in all of the components of the SF-36 and both in the patient- and physician-reported VAS.

Another important finding from the present study was the ability of BFR to increase quadriceps CSA. This is certainly a relevant outcome as muscle atrophy is an important feature in IIM and it is closely related to the sustained disability usually experienced by these patients. The mechanisms by which muscle atrophy occurs in IIM are still not fully understood, but it is thought to involve a vicious circle comprising muscle disuse and muscle waste alongside other factors such as inflammation, metabolic disturbances and steroid myopathy [3,34-36]. The current findings reveal that BFR training may be a suitable therapeutic strategy to offset muscle atrophy in PM and DM, although its underlying mechanisms remain to be fully solved.

Despite the growing number of studies emphasizing the safety aspects of exercise in IIM, there is still empirical concern that exercise could cause disease-flare activity in IIM. In accordance with previous findings [6,7,10,11] BFR training induced neither any alteration in the laboratory markers of safety nor any clinical adverse effects. These findings certainly add to the accumulated body of evidence indicating that exercise training programs are safe for PM and DM patients, extending this notion to BFR training.

This study is not without limitations. The lack of a non-trained control group can be considered as the main limitation, as the natural course of the disease might have influenced the outcomes. However, the short-term nature of this trial, along with the fact that the patients remained in remission, mitigate this possibility. We acknowledge, however, that further randomized controlled trials or a within-subject design with repeated measures would strengthen our findings. The latter would constitute an interesting approach in such a heterogeneous rare disease. Another limitation is the lack of additional markers of disease activity other than muscle enzymes.

Importantly, as this study involved only non-active patients, it is not possible to generalize the present findings to patients with active disease. Further trials with larger and diversified (for example, active, remissive, refractory) samples are necessary in order to advance the knowledge on the effects of BFR in IIM. Likewise, future trials should explore the intrinsic mechanisms by which BFR can improve muscle strength, function, and mass in IIM. Finally, it is imperative to compare the potential of BFR against traditional heavy-load resistance training.

## Conclusion

In summary, we demonstrated that a 12-week supervised low-intensity resistance training program associated with partial blood flow restriction can be safe and effective in improving muscle strength, function, muscle mass, and health-related quality of life in non-active patients with PM and DM.

## Abbreviations

1RM: one-repetition-maximum
AZA: azathioprine
BFR: blood flow restriction
CK: creatine kinase
CP: cyclosporine
CQ: chloroquine diphosphate
CSA: cross-sectional area
CV: coefficient of variation
DM: dermatomyositis
ES: effect size
HAQ: health assessment questionnaire
HCQ: hydroxychloroquine sulphate
HR: heart rate
IIM: idiopathic inflammatory myositis
MMF: mycophenolate mofetil
MRI: magnetic resonance imaging
MTX: methotrexate
PM: polymyositis
POST: after the intervention
PRE: baseline
SF-36: Short form-36 health survey
TST: timed-stands test
TUG: timed up-and-go test
VAS: visual analogue scale
VO_2_: oxygen consumption
VO_2peak_: peak oxygen consumption
